# Association between opioid agonist therapy use and HIV testing uptake among people who have recently injected drugs: a systematic review and meta‐analysis

**DOI:** 10.1111/add.15316

**Published:** 2021-02-03

**Authors:** Claire F. Ferraro, Daniel E. Stewart, Jason Grebely, Lucy T. Tran, Shally Zhou, Carla Puca, Behzad Hajarizadeh, Sarah Larney, Thomas Santo, Julian P. T. Higgins, Peter Vickerman, Louisa Degenhardt, Matthew Hickman, Clare E. French

**Affiliations:** ^1^ Health Protection Research Unit, Population Health Sciences University of Bristol Canynge Hall, 39 Whatley Road, Clifton Bristol BS8 2PS UK; ^2^ National Public Health Speciality Training Programme, South West Bristol UK; ^3^ Kirby Institute, University of South Wales Sydney Sydney Australia; ^4^ National Drug and Alcohol Research Centre University of South Wales Sydney, Sydney, Australia; ^5^ Department of Family Medicine and Emergency Medicine Université de Montréal and Research Centre of the Centre Hospitalier de l'Université de Montréal (CRCHUM) Quebec Canada

**Keywords:** HIV/AIDS, injecting drug users, meta‐analysis, methadone, opioid agonist therapy, systematic review

## Abstract

**Background and aim:**

Globally, nearly one in five people who inject drugs (PWID) are living with HIV, and the rate of new HIV infections in PWID is increasing in some settings. Early diagnosis is crucial for effective HIV control. We reviewed the evidence on the association between opioid agonist therapy (OAT) and HIV testing uptake among PWID.

**Methods:**

We conducted a systematic review searching MEDLINE, Scopus, Web of Science, Cochrane Central Register of Controlled Trials and PsycINFO for studies published from January 2000 to March 2019. Reference lists and conference proceedings were hand‐searched. Observational and intervention studies were eligible for inclusion. Risk of bias was assessed using the Risk of Bias in Non‐Randomised Studies of Interventions (ROBINS‐I) tool. Meta‐analyses were conducted using random‐effects models.

**Results:**

Of 13 373 records identified, 11 studies from Australia, Europe, Malaysia and the United States were included. All studies had at least a serious risk of bias, largely due to confounding and selection bias, making it difficult to draw causal conclusions from the evidence. Ten studies provided data on the association between current OAT use and recent HIV testing. Six showed a positive association, while four provided little evidence of an association: pooled odds ratio (OR) = 1.71, 95% confidence interval (CI) = 1.28–2.27. Looking at having ever been on OAT and having ever been HIV tested, seven studies showed a positive association and three showed either weak or no evidence of an association: pooled OR = 3.82, 95% CI = 2.96–4.95.

**Conclusions:**

Opioid agonist therapy may increase uptake of HIV testing among people who inject drugs, providing further evidence that opioid agonist therapy improves the HIV treatment care cascade.

## Introduction

Globally, there are an estimated 15.6 million people aged 15–64 years who inject drugs (PWID) [1]. Blood‐borne virus infections from injecting drug use are a major contributor to the global burden of disease [2, 3]. There are an estimated 2.8 million PWID living with HIV [1], representing 18% of the global population of PWID and contributing an estimated 4% of disability‐adjusted life years (DALYs) due to HIV [2].

In contrast to an overall 25% decline in global HIV incidence between 2010 and 2017, the incidence among PWID is increasing in some regions [4]. To reverse this trend, there is a need to improve engagement in every aspect of the HIV cascade of care [4]. Early diagnosis, timely linkage to HIV care and initiation of anti‐retroviral therapy (ART) are important, both for the health of the individual and in reducing the risk of onward transmission of HIV [5, 6, 7, 8].

HIV prevention among PWID requires individual, structural and combination approaches [9]. Harm reduction programmes are endorsed by the World Health Organization (WHO) as a key strategy in reducing morbidity and mortality in PWID [4]. Global coverage of harm reduction programmes for PWID improved between 2010 and 2017 [10, 11]. However, in 2017 only approximately half of the 179 countries with evidence of injecting drug use had any form of harm reduction programme [10]. Furthermore, only 34 countries report having targeted programmes for HIV testing among PWID, and the number of PWID receiving HIV tests or accessing ART varies widely [10].

When integrated with HIV services, harm reduction in the form of opioid agonist therapy (OAT) is associated with improved initiation and adherence to ART and viral suppression in PWID living with HIV [12, 13]. OAT is also associated with a reduction in risk of HIV [14] and hepatitis C virus (HCV) acquisition [15]. However, to date, evidence on the association between OAT use and HIV testing uptake has not been synthesized, nor has the strength of the evidence been critically assessed. We conduct the first systematic review and meta‐analysis of the association between OAT use and HIV testing uptake among people who have recently injected drugs. The aims of our review are to assess: (1) the association between current or recent OAT use and HIV testing in the last year; (2) the association between ever having used OAT and ever having been HIV tested; and (3) the risk of bias of the primary studies included in the review.

## Methods

We registered our review protocol with the International Prospective Register of Systematic Reviews (PROSPERO; CRD42019131095). Our report of the review follows the Preferred Reporting Items for Systematic Reviews and Meta‐Analyses (PRISMA) guidelines [16].

### Eligibility criteria

Our review included studies that met the following criteria:

- Population: adults (> 15 years) with a current or recent history of injection drug use (within the last year). We excluded studies in prison populations, as OAT provision in this setting is likely to be significantly different from treatment delivered in other settings.
- Intervention: current or recent (within the last year) OAT use was the primary intervention of interest. OAT use ever was the secondary intervention of interest. We included studies on OAT (both methadone and buprenorphine), irrespective of whether they were delivered in isolation or in conjunction with other harm reduction interventions.
- Comparator: no current OAT use was the primary intervention of interest; no history of OAT use was the secondary intervention.
- Outcome: recent (within the last year) HIV antibody testing was the primary outcome of interest. Ever HIV antibody testing was the secondary outcome.
- Study design: randomized and non‐randomized trials and observational studies with a sample size > 40.

### Search strategy

We searched MEDLINE, Scopus, Web of Science, Cochrane Central Register of Controlled Trials and PsycINFO databases for studies published from January 2000 to March 2019. We used a combination of Subject Headings (e.g. MeSH terms) and free‐text key words. Search terms included HIV, testing, opioid substitution and injecting drug use (see Appendix SI for search strategy). No restriction was placed on language or publication status. We also searched conference abstracts from the Annual Conference on Retroviruses and Opportunistic Infections and the International AIDS Conference for the same period.

We hand‐searched the reference lists of papers selected for inclusion in our review and relevant review papers. Forward citation searches were conducted using Web of Science, Google Scholar and Scopus. We also sought data from known repositories of data on PWID and contacted authors for supplementary data where required.

### Study selection

After the removal of duplicates, titles and abstracts were screened by one reviewer, with 10% checked by a second reviewer. Full texts were retrieved for records identified as potentially eligible and were independently reviewed by two authors using pre‐defined eligibility criteria. Discrepancies were resolved through discussion, with a third reviewer consulted as necessary.

### Data extraction and synthesis

Data were independently extracted by two reviewers into a pre‐defined and piloted Microsoft Excel database. Any discrepancies were identified and resolved. Data extracted included study design, study period, country, method of recruitment, inclusion criteria, sample size, age, gender, OAT type and HIV testing (either self‐reported or documented in medical or other records). Unadjusted and adjusted odds ratios (ORs) and 95% confidence intervals (CIs) were extracted or calculated for each study. Where data were insufficient or missing, we contacted the study authors. Where there was more than one publication from the same study, the one with the most comprehensive and relevant data to our primary outcome was selected as the main information source. Where a manuscript did not present the specific data that we required, but indicated that this may have been collected, this was requested from the study authors.

Statistical heterogeneity was assessed using the Cochran χ^2^ test (*Q*‐test), with the *I* ^2^ statistic used to assess the percentage of variability between studies due to heterogeneity rather than sampling error. Due to the presence of between‐study variability, summary outcomes were calculated using random‐effects meta‐analysis. Prediction intervals were calculated to reflect heterogeneity by indicating the interval within which the true effects of 95% of any similar future studies would be expected to fall [17, 18]. Logit‐transformed outcome estimates were used in all meta‐analyses and the estimates were back‐transformed for reporting. Where available, ORs adjusted for all key confounders throughout the whole study data set were used in the meta‐analyses, otherwise unadjusted estimates were used. A fixed continuity correction of 0.5 was applied where the outcome of a study was 0 or 100%. Meta‐regression was planned to assess factors contributing to heterogeneity across studies. All analyses were conducted using the metan package in Stata version 15 [19].

### Assessment of risk of bias

We assessed the risk of bias using the ROBINS‐I tool (‘Risk of Bias in Non‐randomised Studies – of Interventions’). The tool assesses bias across seven domains: confounding, selection of participants, classification of interventions, deviations from intended interventions, missing data, measurement of the outcome and selection of the reported result. Assessments are made at the outcome rather than study level, so for studies that provided data on both the primary and secondary outcome a risk of bias assessment were conducted for each. Studies were judged as having ‘low’, ‘moderate’, ‘serious’ or ‘critical’ risk of bias for each domain, and the overall risk of bias was then derived. A study was judged as being at low risk of bias overall if it was at low risk of bias for all domains, at moderate risk of bias if it was at a low or moderate risk of bias for all domains, at serious risk of bias if it was at serious risk of bias for at least one domain but not at critical risk of bias in any domain and at critical risk of bias if it was judged to be at critical risk of bias in at least one domain [20].

Risk of bias assessments were undertaken by two reviewers independently with any discrepancies resolved through discussion, and the involvement of a third reviewer as necessary. A priori key confounders were age, gender, time since last injection drug use and markers of severity of addiction (e.g. homelessness, stimulant use in conjunction with injected opioids.)

## Results

### Search results

**Figure 1:**
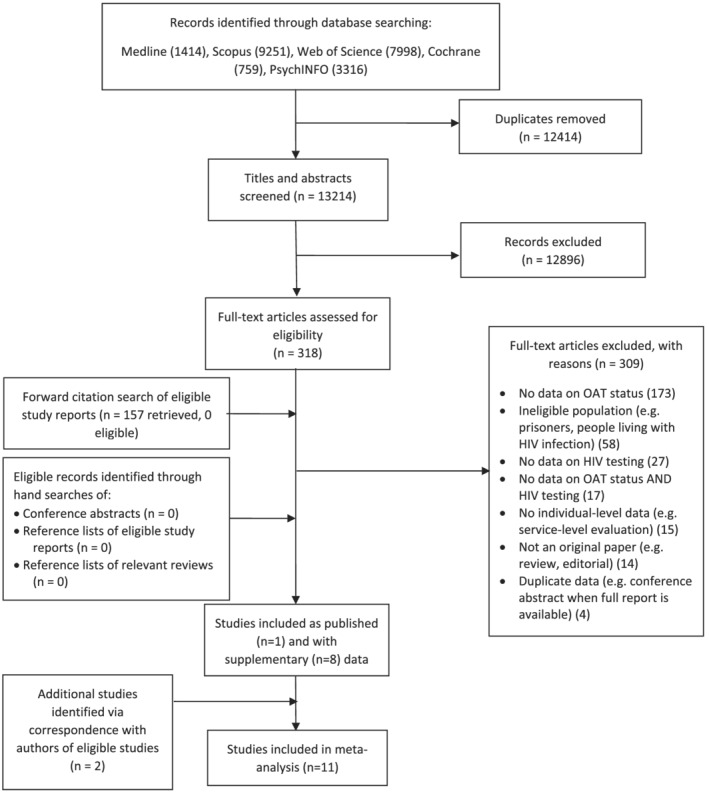
Study selection flow‐chart.

The electronic database searches identified 13 214 records, with a further 159 retrieved from other sources (totalling 13 373). We contacted 69 study authors of the 318 full‐text screened studies to request supplementary data. In total, 11 studies were eligible; one study with data as published [21] and 10 with supplementary data requested from authors [22, 23, 24, 25, 26, 27, 28, 29, 30, 31] (Fig. 1). In four instances, data on OAT use and HIV testing was specifically extracted for inclusion in our meta‐analysis from routinely collected cross‐sectional survey data [26, 32, 33, 34].

All studies were cross‐sectional, except for one randomized controlled trial. The number of participants in each study ranged from 80 to 19 481. A total of 53 012 individuals were included in our analysis, the majority (73%) of whom are male. The mean age of participants within studies ranged from 23.4 to 39.4 years (Table 1).

**TABLE 1:** Description of included studies.

Author, publication year^a^	Study design	Study period	Location	Single/multi‐site^b^	Recruitment	Definition of recent injecting drug use	Total sample size	Demographics	
								No. female (%)	Mean age in years (standard deviation)
Bazazi, 2018 [22]	Cross‐sectional survey	2010	Malaysia	Single centre	Respondent‐driven sampling	Past 1 m	460	10 (3%)^d^	38.6 (9.5)
Bryant, 2012 [23]	Cross‐sectional survey	2009	Australia	Multi‐centre	NSP 2 week period ‘census approach’	Past 1 m	360	125 (35%)	35.8 (9.1)
Dumchev, 2018 [26]	Cross‐sectional survey	2015–2017	Ukraine	Multi‐centre	Respondent‐driven sampling (RDS) seeds were recruited at harm reduction, drug treatment or HIV care sites	Past 1 m	19 481	3465 (18%)^e^	34.9 (8.5)
Guarino, 2015 [27]	Cross‐sectional survey	Not known	USA	Single centre	Purposive sampling strategies: from local drug treatment organizations and former Soviet Union community contacts and respondent‐driven sampling	Past 12 m	80	21 (26%)	23.4 (3.5)
Handanagic, 2016 [21]	Cross‐sectional survey	2014–2015	Croatia	Multi‐centre	Respondent‐driven sampling	Past 1 m	654	151 (23%)	35.8
Harm Reduction Wales (PHW) [34]	Cross‐sectional survey	2016–2019	Wales	Multi‐centre	Convenience sampling: PWID recruited through NSPs nation‐wide	Past 12 m	4273	1182 (28%)	39.4
Makarenko, 2016 [28]	Cross‐sectional survey	2014–2015	Ukraine	Multi‐centre	Respondent‐driven sampling	Past 1 m	1163	275 (24%)	35.4 (8.2)
Metsch, 2012 [30]	Randomized control trial	2009	USA	Multi‐centre	Researchers attempted to approach all patients accessing services at community‐based drug treatment programmes	Past 6 m	264	106 (40%)	38.6 (10.8)
NESI (HPS) [32]	Cross‐sectional survey	2008–2018	Scotland	Multi‐centre	Convenience sampling: PWID attending NSP services	Past 6 m	11 877	3326 (28%)^f^	35.4 (10.3)
UAM (PHE) [33]	Cross‐sectional survey	2013–2018	England, Northern Ireland, Wales	Multi‐centre	Convenience sampling: PWID recruited at drug and alcohol services	Past 12 m	11 775	3137 (27%)^g^	39.4 (8.3)
Williams, 2013 [31]	Cross‐sectional survey	2002–2006	USA	Single centre	Purposive sampling strategies: ethnographers identified specific locations where active PWID could be recruited and interviewed. Mobile Assessment Units were parked adjacent to these identified ‘risk pockets’	Past 3 m^c^	2625	662 (25%)	39 (17–75) (range)

PHW = Public Health Wales; PHE = Public Health England; UAM = unlinked anonymous monitoring; NESI = Needle Exchange Surveillance Initiative; HPS = Health Protection Scotland; NSP = needle and syringe programme; PWID = people who inject drugs;   m = month.

^a^ Public Health Wales, Health Protection Scotland and Public Health England provided data from annual surveys of PWID.

^b^ Multi‐site study defined as PWID recruited from more than one geographical location (e.g. city).

^c^ Williams *et al*. sought to recruit ‘active injecting drug users’; 99% of participants reported injecting during the last 3 months (data provided by authors).

^d^ Demographic data based on sample size of 391 for whom there are complete data.

^e^ Demographic data based on 2017 annual cohort and extrapolated to the total sample size.

^f^ Demographic data only available for aggregate data (2008–18) reporting an HIV test in the last year and receiving methadone in the last 6 months within survey duplicates removed, current PWID (injected in the last 6 months); (*n =* 11 158).

^g^ Demographic data based on 2018 annual cohort and extrapolated to the total sample size.

Studies were based in nine countries: Australia, Croatia, Malaysia, Ukraine, the four countries in the United Kingdom and the United States. Individuals from the United Kingdom (52.7%, *n* = 27 925) and Ukraine (38.9%, *n* = 20 644) comprised the majority of the sample.

### Risk of bias

**Figure 2:**
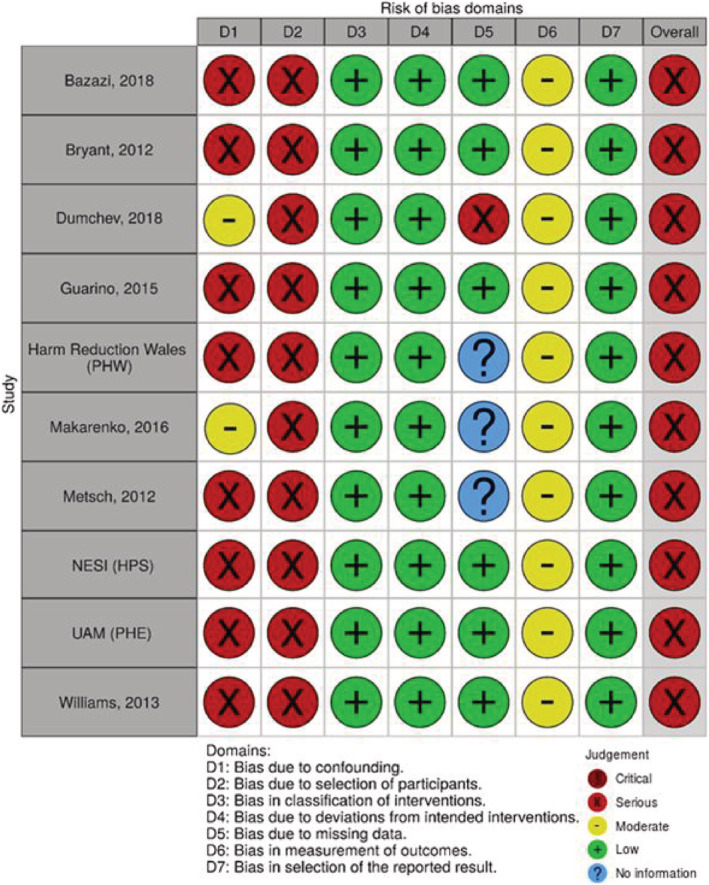
Risk of bias assessments for studies assessing current opioid agonist therapy (OAT) use and HIV testing in the previous year (primary analysis). PHW = Public Health Wales; PHE = Public Health England; UAM = unlinked anonymous monitoring; NESI = Needle Exchange Surveillance Initiative; HPS = Health Protection Scotland. Figure created using robvis [35]. [Colour figure can be viewed at wileyonlinelibrary.com]

**Figure 3:**
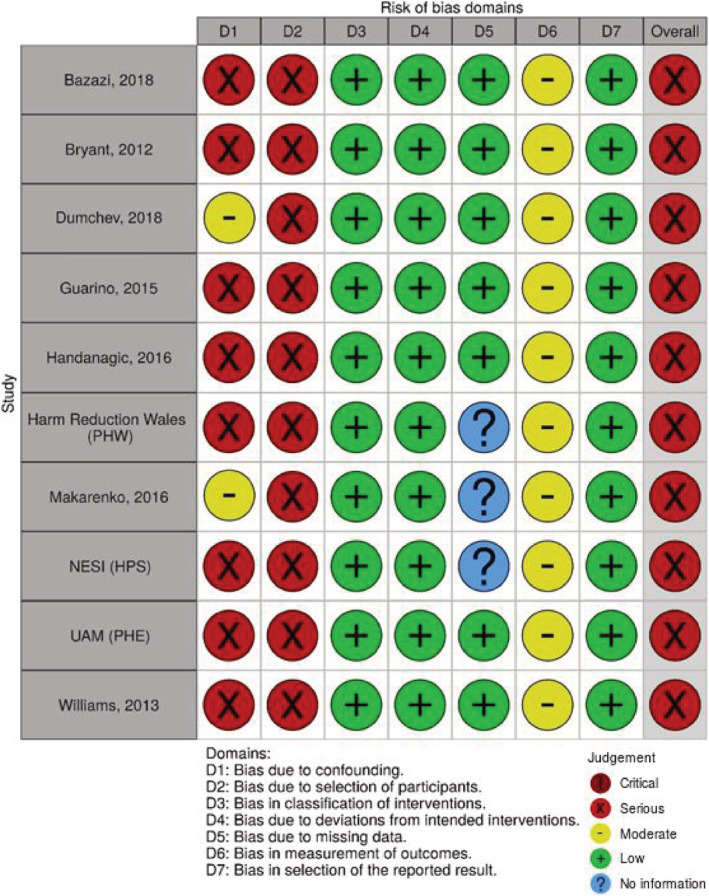
Risk of bias assessments for studies assessing opioid agonist therapy (OAT) use ever and HIV testing ever (secondary analysis). PHW = Public Health Wales; PHE = Public Health England, UAM = unlinked anonymous monitoring; NESI = Needle Exchange Surveillance Initiative; HPS = Health Protection Scotland. Figure created using robvis [35]. [Colour figure can be viewed at wileyonlinelibrary.com]

All studies were assessed as being at serious risk of bias, primarily due to the risk of confounding and selection bias. In the absence of any strong evidence indicating which, if any, factors are likely to confound the association between OAT use and HIV testing we judged studies presenting unadjusted analyses only to be at serious rather than critical risk of bias due to confounding. Two studies provided adjusted estimates for both the primary and secondary analyses and these were assessed as being at moderate risk of bias due to confounding, although at serious risk of bias overall [26, 28]. One study provided adjusted estimates for a proportion of their data [32]. As this study remained at a serious risk of bias and provided similar estimates to the unadjusted data (more information is presented in Appendix S2), we chose to use the larger, unadjusted data to increase the power in our analyses. A further study provided adjusted analyses for the secondary analysis but remained at serious risk of bias due to confounding, as only age and sex were adjusted for [21]. Consequently, it was not appropriate to conduct sensitivity analyses to assess how excluding studies at serious risk of bias influenced the findings. Where there was sufficient information to make an assessment, risk of bias for all other domains was low or moderate (Figs 2 and 3).

### Impact of OAT on HIV testing uptake

**Figure 4:**
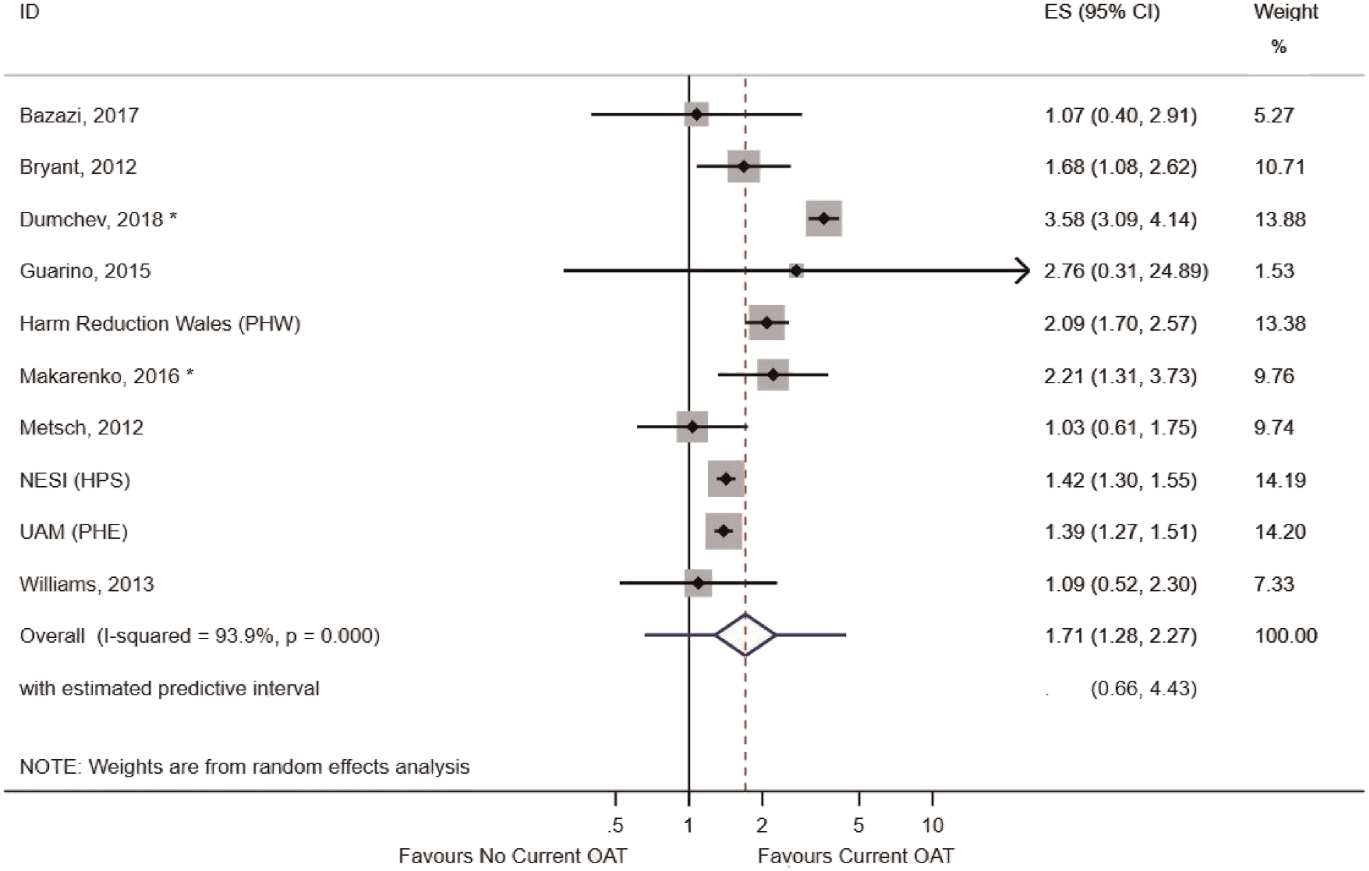
Meta‐analysis of current opioid agonist therapy (OAT) use and HIV testing in the previous year among people who inject drugs (PWID) who have recently injected drugs (primary analysis). PHW = Public Health Wales; NESI = Needle Exchange Surveillance Initiative; HPS = Health Protection Scotland; UAM = unlinked anonymous survey; PHE = Public Health England; OR = odds ratio. *Effect estimates for these two studies are adjusted for age, sex, injection duration, homelessness, injection of cocaine/stimulants and imprisonment. [Colour figure can be viewed at wileyonlinelibrary.com]

Ten studies provided data for our primary analysis: the impact of current OAT use on HIV testing uptake in the previous year (Table 2). Six showed a positive association, while four provided little evidence of an association; ORs ranged from 1.03 to 3.58 across studies. Two adjusted estimates were available for the complete study population and were included in the meta‐analysis [26, 28] (see Appendix S2 for comparisons of unadjusted and adjusted effect estimates). The pooled OR across all 10 studies was 1.71 (95% CI = 1.28–2.27, prediction interval = 0.66–4.43). Inconsistency of results across studies was high (*I* ^2^ = 93.9, *P* < 0.001). No meta‐regression was undertaken due to the small number of studies and the lack of clear candidate factors for explaining heterogeneity (Fig. 4).

**TABLE 2:** Primary and secondary analysis.

Author, publication year^a^	Total sample size^b^	Primary analysis: HIV test in the last year			Secondary analysis: HIV test ever		
		*n*	Current OAT	No current OAT	*n*	Ever OAT	Never OAT
Bazazi, 2018 [22]	460	391	5/33 (15%)	51/358 (14%)	391	61/73 (84%)	248/318 (78%)
Bryant, 2012 [23]	360	320	102/177 (58%)	64/143 (45%)	316	200/228 (88%)	50/88 (57%)
Dumchev, 2018 [26]	19 481	14470^d^	662/892 (74%)	6910/13 578 (51%)	10076^f^	1010/1044 (97%)	6898/9030 (76%)
Guarino, 2015 [27]	80	48	10/11 (91%)	29/37 (78%)	48	15/17 (88%)	28/31 (90%)
Handanagic, 2016 [21]	654	NA	NA	NA	628	130/148 (88%)	297/480 (62%)
Harm Reduction Wales (PHW) [34]	4273^b^	4273	190/399 (48%)	1175/3874 (30%)	4273	1064/1388 (77%)	1214/2885 (42%)
Makarenko, 2016 [28]	1163^c^	1151	33/64 (52%)	323/1087 (30%)	1163	287/315 (91%)	560/848 (66%)
Metsch, 2012 [30]	264	260	79/116 (68%)	97/144 (67%)	NA	NA	NA
NESI (HPS) [32]	11 877	11158^e^	3652/8364 (44%)	986/2794 (35%)	9083^g^	6495/7900 (82%)	633/1183 (54%)
UAM (PHE) [33]	11 775	11 308	3074/8046 (38%)	1005/3262 (31%)	11 308	8031/9851 (82%)	916/1457 (63%)
Williams, 2013 [31]	2625	2568	17/29 (59%)	1433/2539 (56%)	2568	27/29 (93%)	2245/2539 (88%)

PHW = Public Health Wales; PHE = Public Health England; UAM = unlinked anonymous monitoring; NESI = Needle Exchange Surveillance Initiative; HPS = Health Protection Scotland; PWID = people who inject drugs; OAT = opioid agonist therapy; NA = not applicable.

^a^ Public Health Wales, Health Protection Scotland and Public Health England provided data from annual surveys of PWID.

^b^ Total sample size may differ to *n* used in primary and/or secondary analyses due to missing data on OAT use and HIV testing.

^c^ Extent of missing data is unknown. Unpublished data provided only included participants with known data on OAT use status and HIV testing status.

^d^ Primary analysis for Dumchev, 2018 includes data from 2015 and 2017 annual surveys only.

^e^ Aggregate data from the Needle Exchange Surveillance Initiative (NESI) on reporting an HIV test in the last year and receiving methadone in the last 6 months (within survey duplicates removed), current PWID (injected in the last 6 months).

^f^ Secondary analysis for Dumchev, 2018 includes data from the 2017 annual survey only.

^g^ Aggregate data from the Needle Exchange Surveillance Initiative (NESI) on reporting an HIV test in the last year and receiving methadone in the last 6 months (within survey and across survey duplicates removed), current PWID (injected in last 6 months)

**Figure 5:**
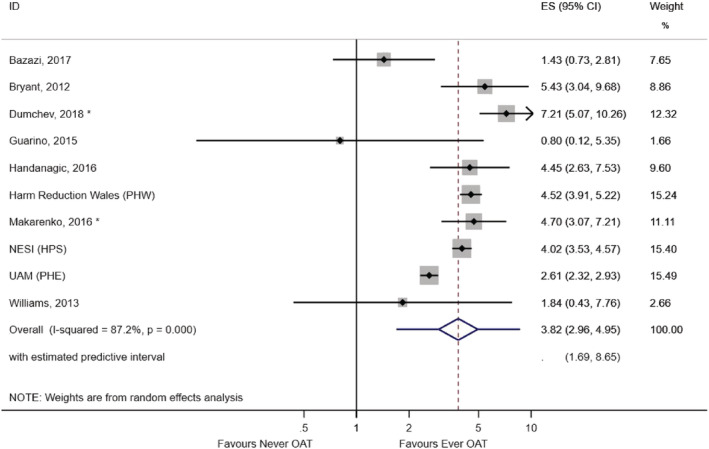
Meta‐analysis of ever opioid agonist therapy (OAT) use and HIV testing ever among people who inject drugs (PWID) who have recently injected drugs (secondary analysis). PHW = Public Health Wales; NESI = Needle Exchange Surveillance Initiative; HPS = Health Protection Scotland; UAM = unlinked anonymous survey; PHE = Public Health England; OR = odds ratio. *Effect estimate for this study is adjusted for age, sex, injection duration, homelessness, injection of stimulants and imprisonment. [Colour figure can be viewed at wileyonlinelibrary.com]

Ten studies provided data for our secondary analysis: the impact of ever using OAT on uptake of HIV testing ever (Table 2). Seven studies showed a positive association and three showed either weak or no evidence of an association. Two adjusted estimates were available for inclusion in the meta‐analysis (Appendix S2). The pooled OR among all studies was 3.82 (95% CI = 2.96–4.95, prediction interval = 1.69–8.65). Heterogeneity was again high (*I* ^2^ = 87.2%, *P* < 0.001) (Fig. 5).

## Discussion

Our comprehensive review found evidence from published and unpublished observational studies and one randomized controlled trial that current OAT use is associated with an increased uptake of HIV testing in the last year among PWID. We also found evidence to suggest that people who had ever taken OAT were more likely to have ever been tested for HIV.

This is the first systematic review, to our knowledge, of the impact of OAT on HIV testing uptake among PWID. Our results are consistent with findings of other studies demonstrating the benefits of OAT use in reducing harms across multiple health outcomes for people who are opioid‐dependent [36]. OAT use has been shown to improve engagement in HIV treatment uptake, adherence and viral suppression, as well as engagement in hepatitis C testing and treatment [12, 37, 38]. OAT use is also associated with a reduced risk of HIV and HCV infections [14, 15].

Reasons for the observed positive association between OAT use and HIV testing uptake are likely to be multi‐factorial. That some OAT programmes require entrants to be HIV tested may be one explanation [39]. The characteristics and preferences of those seeking OAT may also play a role; entry into an OAT programme is indicative of an individual's level of engagement with health‐care providers with regard to drug use and associated health‐care needs. Furthermore, people in OAT will probably also have increased opportunities to be offered HIV testing, although global data on HIV testing programmes for PWID are scarce [10]. Finally, the multiple benefits of OAT use, such as improved functional outcomes [40] and reductions in injecting risk behaviour [39], criminal activity [41], overdose and all‐cause mortality [42], may lead to better engagement in services and a greater readiness to test, and be treated, for HIV.

The major strength of our review is that we included several sources of unpublished data and completed secondary analyses of data that have not previously been applied to this research question. We recognize that publication bias may be an issue—although we screened more than 13 000 study reports for eligibility, there may be relevant data sets that were not identified through our searches because authors had not published data pertaining to OAT use and HIV testing. There is also a small risk that we missed some studies due to lack of complete duplicate screening at title and abstract screening. However, of the 10% of studies screened in duplicate on title and abstract there were no important discrepancies. All studies that reached the full text screening stage were screened in duplicate.

The findings presented in this review are limited by the relatively small number of studies eligible for inclusion. The majority of data in the review originated from either the United Kingdom or Ukraine, which may limit the generalizability of the findings. All studies were conducted in urban areas, and excluded prison populations where drug injecting and HIV testing practices may differ from other settings [43, 44].

Furthermore, the serious risk of bias identified in all the studies included in this review means that findings should be interpreted with caution, and that estimates of association should not be interpreted as convincing evidence of causality. Lack of adjustment for confounding factors, including age, gender, time since last injection drug use, markers of severity of addiction and bias in the selection of participants, were the main reasons that studies were assessed as being at serious risk of bias. However, the three studies providing effect estimates for the primary analysis that were adjusted for all important confounders were similar to the unadjusted estimates, which may suggest that these factors do not strongly influence the association between OAT use and HIV testing.

The temporality of the association between exposure and outcome is also an issue, as the majority (*n* = 10) of studies included in our review were cross‐sectional. We cannot, therefore, assume that OAT use commenced before, rather than after, HIV testing in those studies. To try to minimize this issue we chose current OAT use and HIV testing in the last year as our primary analysis. In future studies it would be interesting and informative to collect data on both the date that OAT started and the date of HIV testing to more clearly understand the temporality of the association.

It was not possible to explore reasons for heterogeneity across the studies in our analysis. It is likely that the characteristics of the study participants and the different settings in which studies were conducted can explain much of the heterogeneity observed. For example, there are variations in both HIV prevalence and OAT coverage by setting. Estimated HIV prevalence among PWID in regions where our included studies were conducted vary between 1.1% (95% CI = 0.8–1.4) in Australasia and 24.7% (95% CI = 15.6–33.9) in eastern Europe [1], while OAT coverage varies from one to two OAT clients per 100 PWID in eastern Europe to 46–95 in western Europe [10].

It was not possible to explore differences in the association between OAT use and HIV testing by gender, due to a lack of disaggregated data. Evidence from some countries suggests that HIV is more prevalent in women who inject drugs than in men [45], and females may face social, structural and psychological barriers to accessing harm reduction services which their male counterparts do not experience [46]. Further studies to explore and address gender differences in the access and outcomes for harm reduction services for PWID would be a welcome addition to the evidence base.

Finally, HIV testing uptake in people who inject stimulants rather than opioids, and consequentially for whom OAT is not indicated, may differ in HIV testing behaviour. This may be due to the different risk factors associated with stimulant, rather than heroin injection, including the sexual transmission of HIV within the men who have sex with men (MSM) community [47]. However, although some PWID in the studies included in our review may be injecting stimulants rather than opioids, a recent systematic review on OAT use and hepatitis C testing and treatment noted that the proportion of PWID who reported ever using opioids was 95–100%, indicating that this issue is unlikely to have substantially biased our findings [38].

For health‐care policymakers and practitioners seeking to reduce the harms associated with injecting drug‐use, the findings of this study provide further support for OAT as a critical component of harm reduction. Together with evidence on the benefits of OAT on the HIV treatment care cascade [12] our findings suggest that, in addition to the benefits of OAT across multiple health domains [36], OAT may also improve HIV testing and treatment among PWID. OAT programmes may also support work in settings where HIV testing programmes for PWID [10] are in development or do not comply with the recommendations made by the WHO [48]. However, even where OAT and HIV testing programmes are well established, there is more work to do to understand barriers and enablers to HIV testing uptake. Recent findings from the United Kingdom, for example, suggest that there are still many PWID who are accessing health and harm reduction services and yet report no history of HIV testing [49].

## Conclusions

Our findings indicate that OAT may increase uptake of HIV testing among PWID, providing further evidence of the benefits of OAT use on the HIV treatment and care cascade.

## Declaration of interests

C.F.F. and D.S. are supported by the South West Public Health Specialty Training Programme. M.H. is supported by the NIHR School for Public Health Research. J.P.T.H. is supported by the NIHR Bristol Biomedical Research Centre. L.D. is supported by an NHMRC Senior Principal Research Fellowship no. 1135991 and by a National Institute of Health (NIH) National Institute on Drug Abuse (NIDA) grant (R01DA1104470). NDARC is supported by funding from the Australian Government Department of Health under the Drug and Alcohol Program. J.G. is supported by a National Health and Medical Research Council Investigator Grant (no. 1176131). The Kirby Institute is funded by the Australian Government Department of Health and Ageing. The views expressed in this publication do not necessarily represent the position of the Australian Government. P.V. acknowledges support from National Institute for Allergy and Infectious Diseases (R01 AI147490) and NIDA (R01DA033679). J.G. reports grants and personal fees from Abbvie, Gilead Sciences, Merck and Cepheid and grants from Hologic and Indivior, outside the submitted work. L.D. reports grants from Indivior and Seqirus, outside the submitted work. S.L. reports grants from Indivior, outside the submitted work. M.H. reports personal fees from Abbvie, personal fees from Gilead Sciences and personal fees from Merck, outside the submitted work. P.V. reports honoraria from Abbvie and Gilead and unrestricted research funding from Gilead. All other authors have no conflicts of interest to declare.

## Author contributions

**Claire Ferraro:** Data curation; formal analysis; investigation; methodology; project administration; validation; visualization. **Daniel Stewart:** Data curation; investigation; methodology; project administration; validation; visualization. **Jason Grebely:** Conceptualization; methodology; supervision. **Lucy Tran:** Data curation; investigation. **Shally Zhou:** Data curation; investigation. **Carla Puca:** Data curation; investigation. **Behzad Hajarizadeh:** Conceptualization; methodology. **Sarah Larney:** Formal analysis. **Thomas Santo Jr:** Formal analysis. **Julian Higgins:** Formal analysis. **Peter Vickerman:** Formal analysis. **Louisa Degenhardt:** Formal analysis. **Matthew Hickman:** Conceptualization; formal analysis; funding acquisition; methodology; supervision. **Clare French:** Conceptualization; data curation; formal analysis; investigation; methodology; project administration; supervision; validation; visualization.

## Supporting information


**Appendix S1.** Search strategy.
**Appendix S2.** Comparison of unadjusted and adjusted effect estimates (where available).
