# Supplementary material for: Association between opioid agonist therapy use and HIV testing uptake among people who have recently injected drugs: a systematic review and meta‐analysis
Source: Addiction. 2021 Feb 3;116(7):1664–76. doi: 10.1111/add.15316 (PMC8248165; doi:10.1111/add.15316)
Supplement: Supplementary file 1 — Appendix S1. Search strategy. Appendix S2. Comparison of unadjusted and adjusted effect estimates (where available). [file ADD-116-1664-s001.docx]

**Appendix I: Search strategy**

**MEDLINE**

| **Search** | **Terms** |
| --- | --- |
| 1 | exp HIV/ or ("human immunodeficiency virus" or HIV).ti,ab. |
| 2 | (opioid substitution* or OST or opioid agonist* or OAT or opioid maintenance* or opiate substitution* or opiate maintenance* or opiate agonist* or methadone therap* or methadone treat* or methadone maintenance* or MMT or buprenorphine therap* or buprenorphine treat* or medication-assisted treat* or medication assisted treat* or MAT or opioid treat* or buprenorphine maintenance* or inject drug* or injecting drug* or injection drug* or drug inject* or drug use* or PWID or IDU or IVDU or drug depend* or substance use* or substance misuse* or substance abuse* or drug addict*).ti,ab. or exp Opiate Substitution Treatment/ or exp Substance Abuse, Intravenous/ |
| 3 | (test or testing or tests or screen* or diagnos*).ti,ab. |
| 4 | 1 and 2 and 3 |
| 5 | limit 4 to yr="2000 -Current" |

**SCOPUS**

| **Search** | **Terms** |
| --- | --- |
| 1 | TITLE-ABS-KEY ( "human immunodeficiency virus" OR hiv ) AND TITLE-ABS-KEY ( "opioid* substitution*" OR ost OR "opioid* agonist*" OR oat OR "opioid maintenance*" OR "opiate* substitution*" OR "opiate* maintenance*" OR "opiate* agonist*" OR "methadone therap*" OR "methadone treat*" OR "methadone maintenance*" OR mmt OR "buprenorphine therap*" OR "buprenorphine treat*" OR "buprenorphine maintenance*" OR "medication-assisted treat*" OR "medication assisted treat*" OR mat OR "opioid treat*" OR "inject* drug*" OR "drug* inject*" OR "drug* use*" OR pwid OR idu OR ivdu OR "drug* depend*" OR "substance* use*" OR "substance* misuse*" OR "substance* abuse*" OR "drug* addict*" ) AND TITLE-ABS-KEY ( test* OR diagnos* OR screen* ) |
| 2 | Limit 2000-current |

**Web of Science**

| **Search** | **Terms** |
| --- | --- |
| 1 | TS=("human immunodeficiency virus" OR hiv) |
| 2 | TS=("opioid* substitution*" OR ost OR "opioid* agonist*" OR oat OR “opioid maintenance*” OR “opiate* maintenance*” OR "opiate* substitution*" OR “opiate* agonist*” OR "methadone therap*" OR "methadone treat*" OR “methadone maintenance*” OR mmt OR "buprenorphine therap*" OR "buprenorphine treat*" OR “medication-assisted treat*” OR “medication assisted treat*” OR MAT OR “opioid treat*” OR “buprenorphine maintenance*” OR "inject drug*" OR "injecting drug*" OR "injection drug*" OR "drug inject*" OR "drug use*" OR pwid OR idu OR ivdu OR "drug depend*" OR "substance use*" OR "substance misuse*" OR "substance abuse*" OR "drug addict*") |
| 3 | TS=(test* OR diagnos* OR screen*) |
| 4 | 1 AND 2 AND 3 (limited to 2000-2018) |

**Cochrane**

| **Search** | **Terms** |
| --- | --- |
| 1 | "human immunodeficiency virus" OR hiv in Title Abstract Keyword AND ((opioid* NEXT substitution*) OR ost OR (opioid* NEXT agonist*) OR oat OR (opioid NEXT maintenance*) OR (opiate* NEXT substitution*) OR (opiate* NEXT maintenance*) OR (opiate* NEXT agonist*) OR (methadone NEXT therap*) OR (methadone NEXT treat) OR (methadone NEXT maintenance*) OR mmt OR (buprenorphine NEXT therap*) OR (buprenorphine NEXT treat*) OR (buprenorphine NEXT maintenance*) OR (medication-assisted NEXT treat*) OR (medication NEXT assisted) OR MAT OR (opioid NEXT treat) OR (inject* NEXT drug*) OR (drug* NEXT inject*) OR (drug* NEXT use*) OR pwid OR idu OR ivdu OR (drug* NEXT depend*) OR (substance* NEXT *use) OR (drug* NEXT addict*)) in Title Abstract Keyword AND (test* OR diagnos* OR screen*) in Title Abstract Keyword - with Publication Year from 2000 to 2018, in Trials (Word variations have been searched)' |

**PsychINFO**

| **Search** | **Terms** |
| --- | --- |
| 1 | ("human immunodeficiency virus" or hiv).mp. |
| 2 | ("opioid substitution*" or ost or "opioid* agonist*" or oat or "opioid maintenance*" or "opiate* substitution*" or "opiate* maintenance*" or "opiate* agonist*" or "methadone therap*" or "methadone treat*" or "methadone maintenance*" or mmt or "buprenorphine therap*" or "buprenorphine treat*" or "buprenorphine maintenance*" or "medication-assisted treat*" or "medication assisted treat*" or MAT or "opioid treat*" or "inject drug*" or "injecting drug*" or "injection drug*" or "drug inject*" or "drug use*" or pwid or idu or ivdu or "drug depend*" or "substance use*" or "substance misuse*" or "substance abuse*" or "drug addict*").mp. |
| 3 | (test* or diagnos* or screen*).mp. |
| 4 | 1 and 2 and 3 |
| 5 | limit 4 to yr="2000 -Current" |

**Appendix II: Comparison of unadjusted and adjusted effect estimates (where available)**

| Author, Publication year | Primary analysis | | Secondary analysis | | Variables adjusted for |
| --- | --- | --- | --- | --- | --- |
|  | Unadjusted OR (95% CI) | Adjusted OR  (95% CI) | Unadjusted OR  (95% CI) | Adjusted OR  (95% CI) |  |
| Dumchev, 2018^a^ | 2.78 (2.38-3.24) | 3.58 (3.09-4.14) | 9.31 (6.56 – 13.22) | 7.21 (5.07-10.26) | Age, sex, injection duration, homelessness ever, injection of cocaine in past 30 days, ever imprisonment. |
| Makarenko, 2016 | 2.52 (1.52-4.18) | 2.21 (1.31-3.73) | 5.27 (3.49-7.97) | 4.70 (3.07-7.21) | Age, sex, injection duration, current homelessness, injection of stimulants in last 30 days, ever imprisonment. |
| NESI^b^ | 1.54 (1.19-1.98) | 1.61 (1.23-2.09) | N/A | N/A | Region, age (per year increase), sex, homeless in last 6 months, injected cocaine in last 6 months, number of times in prison, years since first injection. |

N/A – not applicable (adjusted data not provided); NESI = Needle Exchange Surveillance Initiative; OR = odds ratio; CI = confidence interval

a Primary analysis includes data from 2015 and 2017 annual survey data only. The secondary analysis includes data for 2017 only.

b Adjusted estimates were only available for a sub-set of the data (2017-18) - data presented in this table are therefore for 2017-2018 only
